# Evidence for the evolution of thermal tolerance, but not desiccation tolerance, in response to hotter, drier city conditions in a cosmopolitan, terrestrial isopod

**DOI:** 10.1111/eva.13052

**Published:** 2020-08-19

**Authors:** Aaron R. Yilmaz, Sarah E. Diamond, Ryan A. Martin

**Affiliations:** ^1^ Department of Biology Case Western Reserve University Cleveland Ohio USA

**Keywords:** adaptation, body size, global change, plasticity, urban evolution

## Abstract

Cities are often hotter and drier compared with nearby undeveloped areas, but how organisms respond to these multifarious stressors associated with urban heat islands is largely unknown. Terrestrial isopods are especially susceptible to temperature and aridity stress as they have retained highly permeable gills from their aquatic ancestors. We performed a two temperature common garden experiment with urban and rural populations of the terrestrial isopod, *Oniscus asellus*, to uncover evidence for plastic and evolutionary responses to urban heat islands. We focused on physiological tolerance traits including tolerance of heat, cold, and desiccation. We also examined body size responses to urban heat islands, as size can modulate physiological tolerances. We found that different mechanisms underlie responses to urban heat islands. While evidence suggests urban isopods may have evolved higher heat tolerance, urban and rural isopods had statistically indistinguishable cold and desiccation tolerances. In both populations, plasticity to warmer rearing temperature diminished cold tolerance. Although field‐collected urban and rural isopods were the same size, rearing temperature positively affected body size. Finally, larger size improved desiccation tolerance, which itself was influenced by rearing temperature. Our study demonstrates how multifarious changes associated with urban heat islands will not necessarily contribute to contemporary evolution in each of the corresponding physiological traits.

## 1 INTRODUCTION

Urbanization radically alters the selective landscape for organisms living in cities (Donihue & Lambert, 2014; Johnson & Munshi‐South, 2017). One of the most consistent selective agents within the urban environment is the increase in environmental temperature, largely due to the replacement of vegetation with heat‐retaining impervious surfaces, known as the urban heat island effect (Imhoff, Zhang, Wolfe, & Bounoua, 2010). Urban populations are rapidly evolving in response to these higher temperatures (reviewed in Diamond & Martin, 2020). For example, acorn ants have evolved greater tolerance to high temperatures but reduced tolerance to cold temperatures within multiple cities (Diamond, Chick, Perez, Strickler, & Martin, 2017; Diamond, Chick, Perez, Strickler, & Martin, 2018; Diamond, Chick, Perez, Strickler, & Zhao, 2018). And in water fleas, thermal tolerance along with a suite of life‐history traits have evolved in response to increased water temperatures in urban environments (Brans & De Meester, 2018; Brans, Engelen, Souffreau, & De Meester, 2018; Brans et al., 2017; Brans, Stoks, & De Meester, 2018). But other aspects of the environment besides temperature are also likely to be altered by urban heat islands. In particular, urban heat islands not only contribute to elevated temperature, but along with reduced evapotranspiration, they can also generate drier conditions in cities (Ackerman, 1987; Hass, Ellis, Reyes Mason, Hathaway, & Howe, 2016; Taha, 1997). However, the potential for physiological traits to be altered by the effects of both temperature and aridity in urban environments is largely unknown (but see Andrew, Miller, Hall, Hemmings, & Oliver, 2019; Kaiser, Merckx, & Van Dyck, 2016).

Terrestrial isopods are an excellent system to address the question of multifarious selection and trait evolution in cities. Isopods commonly occur in both urban and nonurban areas (Vilisics, Elek, Lövei, & Hornung, 2007), possess gill structures inherited from their aquatic ancestors, which make them especially sensitive to variation in aridity (Dias et al., 2013; Waterman, 1960), and exhibit physiological adaptation to biogeographic variation in temperature over long evolutionary timescales (Castañeda, Lardies, & Francisco, 2004; Lardies & Francisco, 2008). Laboratory and field studies of terrestrial isopods sampled along latitudinal gradients have demonstrated differences in thermal performance and behavior among populations in response to local selective pressures (Castañeda et al., 2004), as well as evolved differences in reproductive output and metabolic rate among populations along a latitudinal gradient (Lardies & Francisco, 2008). Yet, the potential for contemporary evolution of temperature and desiccation tolerance in warmer, drier environments is unclear. Cities provide an opportunity to explore contemporary evolution in response to multifarious stressors arising from urban heat island effects.

In this study, we performed a common garden experiment to assess evidence for evolutionary divergence in three physiological traits—heat tolerance, cold tolerance, and desiccation tolerance—among urban and rural populations of the terrestrial isopod *Oniscus asellus*. We also explored evidence for evolutionary divergence in body size, which is known to vary across urbanization gradients for a wide array of organisms (Merckx et al., 2018), and also plays a major role in modulating thermal and desiccation tolerance (Chown & Nicolson, 2004). We used multiple urban and rural populations from the greater Cleveland, Ohio, USA area (42°N latitude) with a two temperature rearing design, which additionally allowed us to assess plastic effects of rearing temperature on our focal traits. Specifically, rearing individuals from multiple urban and rural populations under controlled laboratory conditions allowed us to estimate the degree of phenotypic divergence among our traits potentially due to evolved differences between populations and to evaluate the plastic effects of rearing temperature on these traits during development (Diamond & Martin, 2016, 2020; Merilä & Hendry, 2014). It is important to note that because we reared a single generation under standardized laboratory conditions, we can attempt to minimize, but not rule out transgenerational plasticity as an alternative explanation for some of our results.

Based on expectations from previous research on thermal adaptation of ectothermic species in cities (Diamond & Martin, 2020), we predicted that urban populations would evolve higher heat tolerance and diminished cold tolerance compared to rural populations. We further predicted that warmer laboratory rearing conditions would result in a plastic effect of higher heat tolerance and diminished cold tolerance for both urban and rural populations.

Our predictions were more complex for the effect of warmer laboratory rearing conditions on desiccation tolerance. It is currently unknown how responses to temperature and desiccation tolerance interact with one another in *O. asellus*. In ectotherms more generally, physiological responses to different stressors can interact in a variety of different ways from being mutually beneficial to trading off with one another (Chown & Nicolson, 2004). Specifically, in our study system, warmer conditions might prime isopods for responding to desiccation stress and therefore result in higher desiccation tolerance values via cross‐tolerance or a generalized stress response (Gilchrist et al., 2008). Alternatively, warmer conditions might increase energy allocation to heat stress and diminish allocation to desiccation stress, resulting in lower desiccation tolerance (Parkash, Aggarwal, Singh, Lambhod, & Ranga, 2013).

Our predictions were similarly complex for plastic and evolutionary effects of urban warming on body size. We expected that warmer laboratory rearing temperature would contribute to smaller body size via the temperature‐size rule, a widespread pattern of phenotypic plasticity in ectotherms (Sibly & Atkinson, 1994). We further expected the evolution of smaller body size in urban populations based on comparative work in ectotherms across biogeographic clines in temperature (Kingsolver & Huey, 2008). However, these expected patterns might be offset to some degree owing to strong support across terrestrial ectothermic species wherein larger body sizes tend to contribute positively to improved heat tolerance, cold tolerance, and desiccation tolerance (Chown & Nicolson, 2004).

## 2 MATERIALS AND METHODS

### 2.1 Isopod study system and environmental differences among urban and rural habitats

*Oniscus asellus*, the common woodlouse, is a geographically widespread terrestrial isopod (Jass & Klausmeier, 2000). This species is native to Europe but was introduced, possibly as early as 1818 (Say, 1818), to the Americas, where it has since become naturalized and now occurs at high abundance (Leistikow & Wägele, 1999; Wang & Schreiber, 1999). The common woodlouse is found in both undisturbed habitats and human‐altered, urbanized habitats (Vilisics et al., 2007). In both habitat types, this species typically occupies mesic environments with some tree cover where dead wood, including logs and tree bark, are readily available (Brereton, 1957).

**FIGURE 1:**
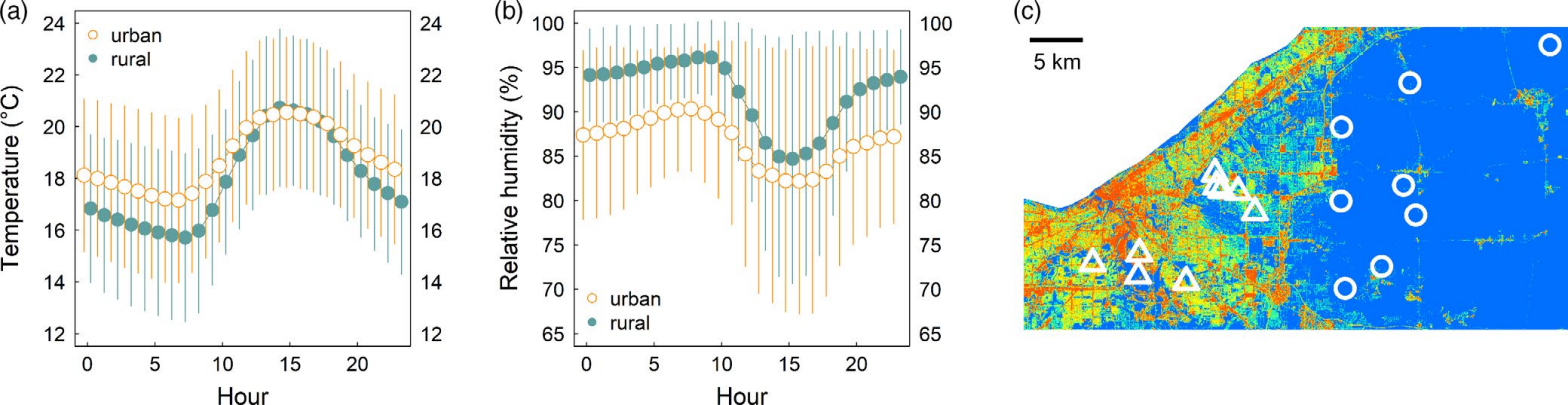
(a) Diurnal plot of temperature (°C) values (mean temperature ± 1 *SD* for each hour of the day) among urban and rural collection sites shown as orange and blue, respectively. (b) Diurnal plot of relative humidity (%) values (mean RH ± 1 *SD* for each hour of the day) among urban and rural sites shown as orange and blue, respectively. Temperature and relative humidity were recorded at thirty‐minute intervals from August to October in isopod refugia. (c) Impervious surface area map of Cleveland, Ohio, USA (data from Elvidge et al., 2007). High ISA values are shown as warm colors and low ISA values are shown as cool colors, with blue designating 0%**–**10% ISA and red designating 90%**–**100% ISA. Rural collection sites are represented by white circles, and urban collection sites are represented by white triangles

Terrestrial isopods have retained the gill structures of their aquatic isopod ancestors and, as a consequence, are highly sensitive to changes in moisture (Dias et al., 2013; Waterman, 1960). Indeed, terrestrial isopods generally forage at night and need to make their water balance back in humid refugia during the day (Hassall, Moss, Dixie, & Gilroy, 2018). Because urban environments are typically both hotter and drier than nonurban (“rural”) environments (Hass et al., 2016; Imhoff et al., 2010), cities might be physiologically challenging for isopods. In Cleveland, Ohio, USA, where we performed our study, the mean growing season temperature difference between urban and rural environments—specifically in microsites where *O. asellus* were collected—is over 1°C (mean ± *SD*: urban = 18.79°C ± 3.29; rural = 17.67°C ± 3.31), with a pattern of nighttime‐biased warming (Figure 1a). Furthermore, the relative humidity between these same sites also differs, with rural sites exhibiting greater relative humidity than urban sites (Figure 1b, mean ± *SD*: urban = 86.6 RH ± 11.26; rural = 94.02 RH ± 8.48). Notably, when subjected to a constant RH below 94%, this species dies within 48 hr (Wright & Machin, 1990). We recorded temperature and humidity by placing data loggers (iButton DS1923) at four rural and three urban Cleveland sites. We had one logger for each site, suspended inside open‐ended PVC tubes with garden wire and were left out in the field from late August to mid‐October of 2019, and set to record temperature and humidity at thirty‐minute intervals. Loggers were placed within microhabitats where isopods had been collected at a given site (e.g., under logs or fallen bark).

### 2.2 Isopod collections and common garden experiment

We collected *O. asellus* from May until July of 2018 from nine urban and eight rural populations across Cleveland, Ohio, USA (Figure 1c, Table S1). Populations inhabiting sites with more than 50% impervious surface area (ISA) were considered urban, and rural populations were defined as those inhabiting sites with less than 20% ISA (measured at 1 km resolution, Elvidge et al., 2007). We did not collect from sites with ISA between 20% and 50% ISA. Isopods were collected from under tree bark, logs, and rocks within urban forest fragments or rural woodlands. The average number of isopods (±1 *SD*) we collected at each site was 5.94 ± 2.49. These were divided among several 120‐ml plastic containers and were housed in a laboratory at Case Western Reserve University. The average number of isopods per container (±1 *SD*) was 4.06 ± 0.429 (Table S1), and each of these containers of isopods was designated as a “replicate.” Each container had a base of plaster of Paris mixed with charcoal (10:1), which was moistened with distilled water regularly throughout the experiment. The plaster base provided isopods with a consistently high relative humidity between 96% and 100%. Thus, although relative humidity differs between urban and rural sites in the field (Figure 1b), we effectively kept humidity constant within the laboratory growth chambers. This design allowed us to explore the plastic effect of temperature on desiccation tolerance, as opposed to the plastic effect of humidity on desiccation tolerance. We provided each container of isopods with approximately 1.0 g of fresh carrot and a 50/50 mix of leaf litter from the urban and rural collection sites. We replenished these food resources approximately every 6 days (the average ± 1 *SD* number of days between feedings was 6.18 ± 2.01).

We acclimated the isopod replicates for at least 48 hr in a growth chamber (DigiTherm DT2‐MP‐47L) maintained at a constant 25°C before haphazardly assigning them to growth chambers (Percival 36‐VL) maintained at either a high (29°C) or low (21°C) temperature treatment. We performed the haphazard assignment in all instances except where there were two replicates from the same site, in which case we assigned one replicate to the high temperature treatment and the other to the low temperature treatment (only one site out of 17 had more than one replicate in a given temperature treatment, Table S1). The two temperature treatments (29 and 21°C) each had a diurnal temperature shift, where the nighttime temperature was decreased by 5°C. The temperature shift was synced to the photoperiod (a long daylight cycle, 14L:10D). These temperature treatments, respectively, are near the daytime mean average temperature (21°C) and the highest temperature (29°C) reached in both urban and rural refugia (Figure 1a). The environmental temperature measurements were conducted contemporaneously with the experiment, and the rearing temperature treatments were modified from an early study (Diamond, Chick, Perez, Strickler, & Zhao, 2018). Although these treatments did not incorporate the nighttime‐biased warming measured in the field, they were chosen to measure the developmental plasticity of our focal traits in response to temperature, rather than to recreate the natural thermal environment.

An F1 generation of isopods was produced within the temperature treatments from field‐caught females. This allowed us to mitigate field developmental acclimation effects as well as mitigate potential transgenerational effects (e.g., maternal effects) in the offspring. These F0 females were either already carrying brood when brought into the laboratory (i.e., the pregnancies were the result of mating and fertilization in the field) or became pregnant while being reared in the laboratory (i.e., either as a result of mating or by using stored sperm). Within approximately seven days after birth, each individual clutch of F1 isopods was removed from its parent replicate and given its own container and replicate designation. Since each parent replicate consisted of multiple F0 females, the F1 offspring within a given replicate were not necessarily all siblings (i.e., it is possible that two or more F0 females contributed offspring to an F1 replicate since multiple pregnant F0 females were housed in each parent replicate container). The rearing protocol for the F1 offspring was identical to that of the parental generation, and the offspring remained in the temperature treatment of their parents (see Figure S1 for a diagram of the rearing design).

We reared the F1 replicates for a minimum of 80 days up to 189 days before they were assayed for thermal tolerance and desiccation tolerance. It typically takes this species around three months under laboratory conditions to reach sexual maturity (Rigaud, Moreau, & Juchault, 1999). Variation in collection dates and laying times among field‐collected isopods, along with logistical constrains for the timing of the thermal tolerance and desiccation tolerance trials, resulted in variation in the number of days individual replicates spent in the rearing treatments. The average (±1 *SD*) number of days F1 isopods spent in the temperature treatments was 133 ± 22.9. However, the number of days spent in the rearing treatments did not differ significantly by source population (*F*_1,23.58_ = 1.967, *p* = .174), temperature treatment (*F*_1,24.58_ = 0.133, *p* = .719), or in the interaction between source habitat and temperature treatment (*F*_1,23.58_ = 0.0057, *p* = .940) tested with a linear mixed effects model with replicate as a random effect. Furthermore, the number of days spent in the rearing treatments did not affect thermal tolerance or desiccation tolerance (Table S6), tested with separate linear mixed effects models with replicate as a random effect. The number of days spent in the rearing treatments did influence body size (see Section 3 and Table 1).

**TABLE 1:** Statistical model summaries for body size responses in field caught and laboratory‐reared isopods

Response	Term	Estimate	*SE*	*F*	*df*	*p*
Field‐caught body mass	Source population	−0.005	0.008	0.453	1,6.085	0.526
Laboratory‐reared body mass	Days reared	0.147	0.011	186.492	1,482.95	**2.2E−16**
	Rearing temperature	6.613	1.607	16.942	1,23.85	**4E−4**
	Source population	−0.744	1.601	0.216	1,24.21	0.646

Note

Estimates, standard errors, *F* test statistics, degrees of freedom, and *p‐*values for the significance are reported. Significant *p‐*values at the .05 level are indicated in bold font.

### 2.3 Thermal tolerance

We assessed both the critical thermal maximum (CT_max_) and the critical thermal minimum (CT_min_) of individual F1 isopods. CT_max_ and CT_min_ were both defined as the temperature at which an isopod could not right itself for at least 10 s after being positioned onto its back (Lutterschmidt & Hutchison, 1997). We selected replicates with at least ten individuals for thermal tolerance assays (Table S3). The temperature treatments and source populations were unknown to the observer for these trials. We placed individuals into 1.5‐ml Eppendorf tubes and haphazardly assigned half of these individuals to be tested for CT_max_ and the other half for CT_min_. Individuals were not tested for more than a single measure of tolerance (thermal and desiccation) due to the destructive nature of the testing. We used a dynamic temperature ramping protocol (Terblanche et al., 2011) with a dry block incubator (Boekel Scientific Tropicooler) to assess CT_max_ and CT_min_. For CT_max_, isopods were acclimated to 34°C for five minutes, and the temperature was then increased by 1°C every minute until isopods could not right themselves within 10 s of gentle tapping on the tube. Likewise, CT_min_ was assessed by acclimating the isopods at 16°C for five minutes and then decreasing the temperature by 1°C every minute until isopods could not right themselves within 10 s of gentle tapping on the tube.

### 2.4 Desiccation tolerance

We used a benchtop desiccator (Bel‐Art “Space Saver” 0.09 cu. ft.) to assess the desiccation tolerance of individual F1 isopods to a static, low relative humidity for both urban and rural habitats, and both high and low temperature treatments (Table S2). The temperature treatments and source populations were also unknown to the observer for these trials. For desiccation tolerance, we selected replicates with at least five individuals in order to be consistent with the sample size used for each thermal tolerance test. As the desiccation tolerance testing took place after the thermal tolerance tests, a combination of isopod mortality and use of isopods in the thermal tolerance assays meant that some, but not all, of the replicates overlapped between the thermal and desiccation tolerance testing (Table S3). As noted above, individual isopods were only tested for a single tolerance measure. Isopods selected for desiccation tolerance were housed without food in their original containers for a period of 48 hr prior to testing. To ensure a relative humidity of ~33%, we placed a MgCl_2_ salt solution in the base of the desiccator before assessing desiccation tolerance (Young, 1967). We massed individual isopods and placed them into labeled 1.8‐ml cryovials with a moist cotton ball plug in the top. We then placed the vials in the desiccator, removed the plugs, and visually checked the isopods every 10 min, and physically checked them every 30 min. The 10‐min checks consisted of observing the isopods through the clear lid of the desiccator; isopods were scored as having reached their desiccation tolerance if they were unable to right themselves within 10 s. The 30‐min checks consisted of removing the vials from the desiccator and positioning the isopods onto their backs by inverting the tube; if an isopod could not right itself within 10 s of gentle tapping on the tube, it was scored as having reached its desiccation tolerance.

### 2.5 Body size

Because body size itself can respond to urban heat island effects and can also influence both thermal and desiccation tolerance (Chown & Nicolson, 2004), we evaluated both of these possibilities. In all assessments of body size, we measured the wet mass of individual isopods on a digital balance with a precision of 0.0001 g (Sartorius MSE124S‐100‐DA). We first explored whether body size differed between the common garden‐reared urban and rural source population isopods, and across the two laboratory rearing temperatures. In addition, we explored whether isopod body size differed between field‐caught urban and rural populations. After exploring the effects of temperature on body size, we then examined the relationship between body size and each of the thermal tolerance and desiccation tolerance traits.

### 2.6 Statistical analyses

We performed all statistical analyses using R 4.0 (R Core Team, 2020). We constructed a series of models to evaluate the mechanisms underlying phenotypic shifts in body size, thermal tolerance traits, and desiccation tolerance. Specifically, we aimed to quantify whether isopods exhibited evidence for plastic (i.e., a response to rearing temperature within urban and rural populations) and evolved (i.e., mean differences between urban and rural populations) responses to temperature in body size, thermal tolerance, or desiccation tolerance. All linear mixed effects models were fit using restricted maximum likelihood (“lme4”) (Bates et al., 2015), and the significance of the fixed effects was tested using the Satterthwaite denominator degrees of freedom approximation (“lmerTest”) (Kuznetsova, Brockhoff, & Christensen, 2017).

We first asked if body size (i.e., wet mass) differed between urban and rural populations in the field‐caught generation using a mixed effects model with source population as our fixed effect (whether the population was sourced from an urban or rural site) and collection site as a random effect. We next used a separate linear mixed model to evaluate body size in the common garden experiment with the main effects of source population, rearing temperature, and the interaction of source population and rearing temperature. We also included a covariate for the number of days spent in the rearing treatments. The interaction between source population and rearing temperature was nonsignificant and was dropped from the final model (*F*_1,22.81_ = 1.281, *p* = .269). We included replicate as a random effect to account for nonindependence of isopods reared within the same container.

To analyze CT_max_ and CT_min_, we fit separate linear mixed effects models with the focal response variable and the main effects of source population, rearing temperature, the interaction of source population and rearing temperature and a covariate for body size. The interactions between source population and rearing temperature were nonsignificant and were dropped from the final models (CT_max_: *F*_1,16.390_ = 0.158, *p* = .696; CT_min_: *F*_1,16.022_ = 0.342, *p* = .567). We again included replicate as a random effect to account for nonindependence of isopods reared within the same container. We fit a linear model to analyze tolerance breadth—the difference between CT_max_ and CT_min_—because tolerance breadth was calculated for each replicate (mean CT_max_–mean CT_min_). We again included the main effects of source population, rearing temperature, the interaction of source population and rearing temperature and a covariate for body size. The interaction between source population and rearing temperature was nonsignificant and was dropped from the final model (*F*_1,15_ = 0.513, *p* = .485).

For desiccation tolerance, exploratory data analysis suggested that both direct and indirect effects of rearing temperature and body size potentially influenced tolerance. Specifically, rearing temperature had a marginally nonsignificant (*F*_1,18.441_ = 3.902, *p* = .063) effect in a mixed effects model (with replicate as the random effect) that also included the nonsignificant effect of source population and the highly significant effect of body size (Table S5). However, the effect of rearing temperature was not present when body size was dropped from the model (*F*_1,17.147_ = 0.964, *p* = .340, Table S5), suggesting that the effect of rearing treatment on desiccation tolerance is only apparent after accounting for the direct effect of body size (Figure S2). To better understand these effects, we employed confirmatory structural equation modeling using the *piecewiseSEM* library (v 2.0.2, Lefcheck, 2016) along with the *lme4* library to account for nonindependence of isopods reared within the same container by including replicate as a random effect. We constructed a model with hypothesized direct and indirect pathways influencing desiccation tolerance. We included individual direct paths between rearing temperature, source population, and body size to desiccation tolerance and two indirect paths with rearing temperature and source population each influencing desiccation tolerance through their effects on body size. We standardized each predictor to a mean of zero and standard deviation of one for direct comparison between effects.

## 3 RESULTS

**FIGURE 2:**
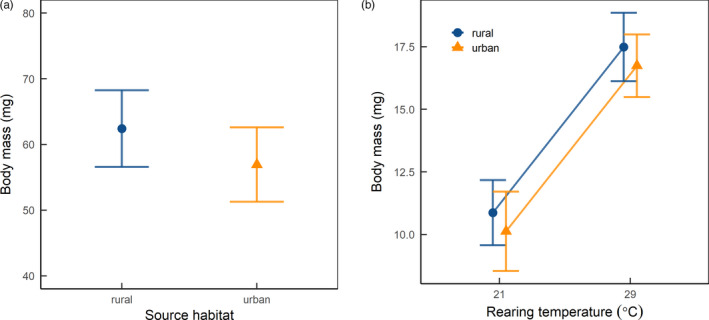
Results for: (a) body mass (mg) as a function of source habitat for field‐caught isopods, and (b) body mass (mg) as a function of temperature treatment across urban and rural populations for laboratory‐reared isopods. Predicted values ± 1 *SE* from linear mixed effects models are shown, with the urban population in triangle symbols and the rural population in circles

Body size did not differ between urban and rural populations in the field‐caught isopods (Table 1, Figure 2a). For the F1 generation, body size differed between rearing treatments (Table 1) with isopods in the warmer rearing treatment reaching 65% larger body size on average (Figure 2b). In addition, body size was positively related to the number of days spent in the rearing treatment (Table 1).

**FIGURE 3:**
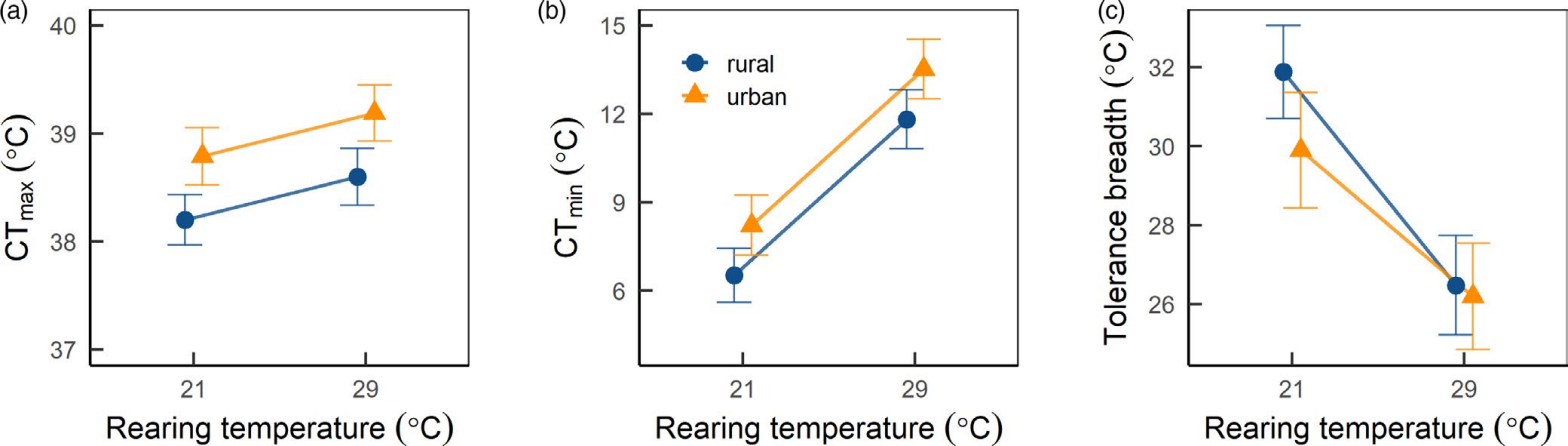
Thermal tolerance responses across urban and rural populations reared under two temperature treatments. Results for: (a) CT_max_ (critical thermal maximum, °C), (b) CT_min_ (critical thermal minimum, °C), and (c) thermal tolerance breadth (CT_max_–CT_min_, °C) as functions of rearing temperature (21 and 29°C). Predicted values ±1 *SE* from linear mixed effects models are shown, with the urban population in triangle symbols and the rural population in circles

We next asked whether CT_max_, CT_min_, or thermal tolerance breadth exhibited results consistent with evolved and/or plastic responses. Results for CT_max_ suggest an evolved higher CT_max_ in response to urbanization (*p = *.052, Figure 3a, Table 2). However, we did not detect a plastic response of CT_max_ to rearing temperature for either urban or rural populations (Figure 3a, Table 2). For CT_min_, we did not detect an effect of evolutionary divergence between urban and rural populations (Figure 3b, Table 2). We did find evidence for a plastic effect of rearing temperature on CT_min_, with lower rearing temperature conferring improved tolerance of cold temperatures (Figure 3b, Table 2). Driven predominantly by the lack of evolutionary divergence in CT_min_, we likewise found no evidence of evolutionary divergence in thermal tolerance breadth between urban and rural source populations, though we again found a significant plastic effect of rearing temperature on thermal tolerance breadth (Figure 3c, Table 2). Specifically, isopods reared at the lower rearing temperature had broader thermal tolerance ranges than those raised at the higher temperature (Figure 3c, Table 2). There was no effect of body size for any thermal tolerance trait (Table 2).

**TABLE 2:** Statistical model summaries for thermal tolerance responses

Response	Term	Estimate	*SE*	*F*	*df*	*p*
CT_max_	Body mass	−18.214	20.206	0.813	1,120.554	0.369
	Rearing temperature	0.399	0.315	1.601	1,22.872	0.218
	Source population	0.589	0.284	4.32	1,17.308	0.052
CT_min_	Body mass	−11.536	84.362	0.019	1,104.303	0.891
	Rearing temperature	5.296	1.240	18.252	1,23.316	**2.8E−4**
	Source population	1.702	1.079	2.489	1,16.590	0.134
Thermal breadth	Body mass	−28.382	153.958	0.034	1,16	0.856
	Rearing temperature	−4.760	1.608	8.765	1,16	**0.009**
	Source population	−1.118	1.153	0.940	1,16	0.347

Note

Estimates, standard errors, *F* test statistics, degrees of freedom, and *p‐*values for the significance of source population, rearing temperature and the covariate of body mass are reported. Significant *p‐*values at the .05 level are indicated in bold font.

**FIGURE 4:**
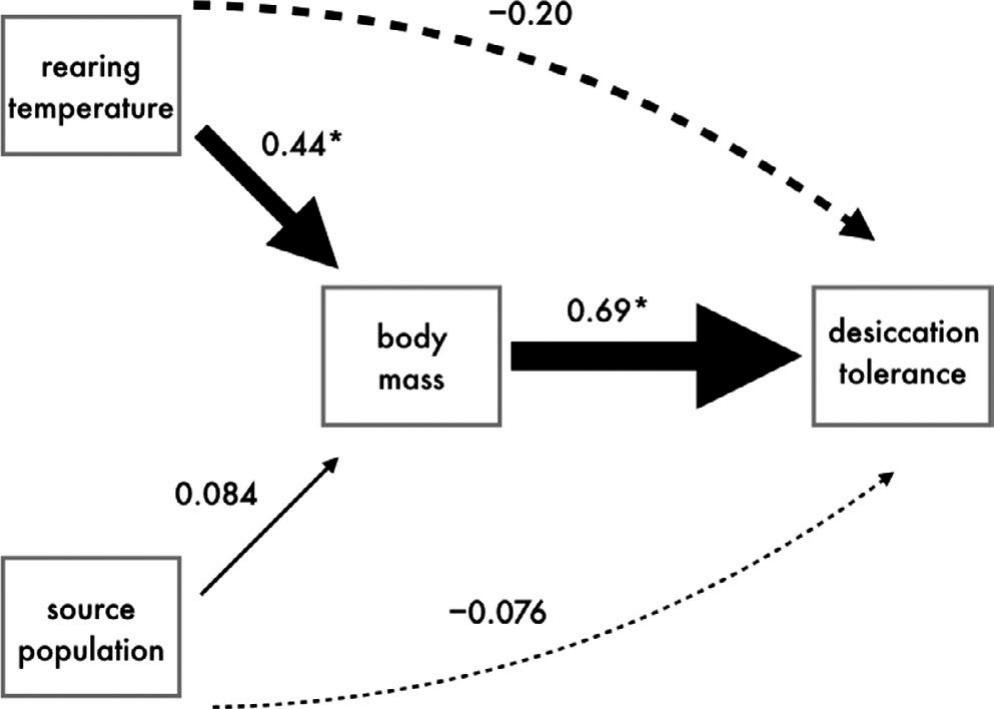
Structural equation model of how body mass, rearing temperature, and source population affect desiccation tolerance. Solid lines indicate positive paths and dashed lines indicate negative paths. Line width is proportional to the standardized path coefficient shown above each line. Asterisks indicate significant paths at the 0.05 level

Finally, our path analysis for desiccation tolerance revealed a direct positive effect of body size, with greater tolerance in larger isopods (Figure 4, Table 3). In contrast, source population had weak, nonsignificant effects on desiccation tolerance and body size, and consequently, there is no evidence that these traits have diverged between urban and rural populations (Figure 4, Table 3). Interestingly, we found contrasting direct and indirect effects of rearing temperature on desiccation tolerance. First, rearing temperature had a positive indirect effect on tolerance acting through body size, where the warmer rearing temperature of 29°C resulted in greater body size (Figure 4, Table 3). However, there was also a weaker, direct negative effect of rearing temperature on desiccation tolerance itself, although this path was nonsignificant (Figure 4, Table 3). Consequently, differences in desiccation tolerance between rearing temperatures are only manifest when comparing isopods of the same body size (Figure S2). Isopods reared at 29°C tended to reach greater size, which increases desiccation tolerance, but isopods reared at 21°C had greater desiccation tolerance for a given body size (Figure S2). Calculating the relative strengths of these direct and indirect effects of rearing temperature revealed that the direct negative effect of rearing temperature on tolerance was almost half the indirect effect acting through body size (direct path: −0.197, indirect path: 0.441 × 0.869 = 0.304, Table 3, Figure 4).

**TABLE 3:** Statistical summaries for the structural equation model of desiccation tolerance including estimates, standard errors, critical values, *p‐*values, and standardized estimates for each path

Path	Estimate	*SE*	*df*	Critical value	*p*	Std. estimate
Rearing temperature → Body mass	5.932	1.973	17	3.006	**0.008**	0.441
Source population → Body mass	1.136	1.983	17	0.573	0.574	0.084
Body mass → Desiccation tolerance	5.694	0.661	146	8.619	**<0.0001**	0.689
Rearing temperature → Desiccation tolerance	−21.894	11.084	17	−1.975	0.065	−0.197
Source population → Desiccation tolerance	−8.442	10.449	17	−0.808	0.430	−0.076

Note

Significant *p‐*values at the .05 level are indicated in bold font.

## 4 DISCUSSION

Environments impose multifarious selection on populations (Riesch, Martin, & Langerhans, 2019; Schluter, 2000), but how multiple traits respond to multiple axes of environmental variation remains an important question in ecology and evolution (Sih, Ferrari, & Harris, 2011). Ongoing anthropogenic changes make this question all the more timely. Cities, as an ever‐growing anthropogenic disturbance, alter environments in many ways. Two of the key axes of variation involve temperature and aridity (Ackerman, 1987; Hass et al., 2016; Imhoff et al., 2010; Taha, 1997). Specifically, cities are often hotter and drier than surrounding undeveloped areas (Ackerman, 1987; Hass et al., 2016; Imhoff et al., 2010; Taha, 1997). We used a terrestrial isopod to explore the potential for contemporary evolution of temperature tolerance, desiccation tolerance, and body size to hotter, drier conditions in cities. Although our results suggest that urban isopods may have evolved higher heat tolerance, desiccation tolerance did not evolve. Cold tolerance, while highly plastic in response to temperature for both urban and rural populations, did not show an evolved loss of cold tolerance in urban populations. Body size also showed no evidence of evolutionary divergence, though it did plastically modulate the desiccation tolerance response. Most importantly, our results lend further empirical support to the growing pattern of urban evolution of heat tolerance across a diverse suite of organisms. In addition, our study highlights the fact that other mechanisms and responses, such as plasticity for cold tolerance and a lack of evolutionary divergence in desiccation tolerance, are possible in urban environments. Thus, while cities might differ from undeveloped areas along multiple axes, whether contemporary evolution occurs would appear to depend on the trait and stressor under consideration.

Cities are increasingly being used to address the question of parallel or convergent evolution, that is, whether traits evolve similarly in response to a common agent of selection (Rivkin et al., 2019). In this regard, our study adds support to the parallel evolution of higher heat tolerance under urban heat islands. We found an increase in heat tolerance on the order of 0.6°C between urban and rural populations of the terrestrial isopod, *Oniscus asellus* in our common garden experiment (Figure 3a, Table 2). Acorn ants also exhibit increased heat tolerance in two out of three cities tested in eastern North America (Diamond, Chick, Perez, Strickler, & Zhao, 2018), and *Daphnia magna* shows repeated evolution of higher heat tolerance in multiple populations across cities in Flanders, Belgium (Brans et al., 2017). Although we are still in the early stages of amassing sufficient data on the evolution of heat tolerance in urban heat islands, the pattern does seem to be a consistent one thus far, particularly compared with other traits such as body size (Merckx et al., 2018; Yilmaz et al., 2019). Indeed, the parallel contemporary evolution of heat tolerance in cities is especially interesting as a counterexample to the broad range of studies that measure heritability of physiological traits and find evidence of limited evolutionary potential (Diamond, 2016; Hangartner & Hoffmann, 2016; Kellermann, Van Heerwaarden, Sgrò, & Hoffmann, 2009; Schou, Kristensen, Kellermann, Schlötterer, & Loeschcke, 2014).

As an important caveat, while our common garden design mitigates potential parental effects, it does not exclude them or other sources of transgenerational plasticity (e.g., grandparental effects) as an alternative explanation for the suggestive divergence in heat tolerance we found between urban and rural populations. Field‐caught females were kept in the laboratory so that their brooding offspring could develop in the temperature treatments. However, we were unable to solely use captive‐bred isopods in our study or rear multiple generations in the laboratory. Therefore, it is possible that shifts in thermal tolerance between urban and rural populations were at least partly due to transgenerational plasticity induced by the natal environment of the field‐caught adults. Although similar divergence in thermal tolerance between urban and rural acorn ants does not appear to be explained by parental effects (Martin, Chick, Yilmaz, & Diamond, 2019), and parental effects tend to decline quickly through ontogeny (Moore, Whiteman, & Martin, 2019), this possibility should be further explored for *O. asellus*.

While the isopods exhibit similar responses to other species with respect to evolutionary divergence in heat tolerance in cities, they differ markedly from these species in their plasticity in heat tolerance. In general, ectotherms increase their heat tolerance in response to rising temperatures (Angilletta, 2009; Chown & Nicolson, 2004), though many researchers have noted that the amount of plasticity is still relatively small compared with other traits such as cold tolerance, body size, and growth rate (Gunderson & Stillman, 2015; Sørensen, Kristensen, & Overgaard, 2016). The isopods we tested did not exhibit a significant response to laboratory rearing temperature for either the rural or urban populations (Figure 3a, Table 2). As a consequence, isopods appear to be a relatively unique case where the response to temperature is purely a canalized, and potentially evolved response to urban heat islands, absent any detectable phenotypic plasticity. Indeed, this result contrasts with the plastic responses of other invertebrates to urbanization including acorn ants and *Daphnia magna* which display higher heat tolerance under warmer laboratory rearing conditions (Brans et al., 2017; Diamond et al., 2017; Diamond, Chick, Perez, Strickler, & Martin, 2018; Diamond, Chick, Perez, Strickler, & Zhao, 2018).

The isopods also exhibit a unique cold tolerance response to the urban heat island, with a purely plastic response of cold tolerance to rearing temperature such that warmer conditions led to diminished cold tolerance (Figure 3b, Table 2). This result is consistent with those from many other ectothermic species (Angilletta, 2009; Chown & Nicolson, 2004). However, we do note that there was a trend toward diminished cold tolerance of the urban population isopods compared with the rural population isopods (Figure 3b, Table 2). Although the magnitude of divergence was appreciable, on the order of 1.7°C, due to substantial variation in cold tolerance responses among individuals, the population divergence was nonsignificant, and other methods might be needed to more accurately assess cold tolerance. There is considerable debate in the literature about whether the relatively greater variation in cold tolerance is biologically real or an artifact of being able to measure the loss of muscular coordination or righting ability at cold temperatures (Addo‐Bediako, Chown, & Gaston, 2000; Terblanche et al., 2011). Assessing LT_50_ under different cold temperature regimes or thermolimit respirometry might enable us to resolve this question in the isopod system (e.g., Lighton & Turner, 2004). Other arthropods such as acorn ants do show an evolutionary loss of cold tolerance in urban environments (Diamond et al., 2017; Diamond, Chick, Perez, Strickler, & Martin, 2018; Diamond, Chick, Perez, Strickler, & Zhao, 2018), though white clover typically shows the opposite trend of evolved improvement in cold tolerance in cities due to snow removal and exposure to cooler air temperatures (Thompson, Renaudin, & Johnson, 2016). It is unclear why, for the acorn ant system, cold tolerance is diminished in the city, though genetic correlation with heat tolerance and energy allocation tradeoffs are possible (Diamond, Chick, Perez, Strickler, & Zhao, 2018). In general, responses to cold stress in the city therefore appear to be complex, though further study of the isopod system could potentially help resolve some of this complexity.

Perhaps our most surprising result was the lack of evolution of desiccation tolerance in urban isopods. While isopods have evolved impressive adaptations to terrestrial life, their desiccation tolerances remain well below those of other arthropods such as insects or arachnids (e.g., Broly, Devigne, & Deneubourg, 2015). Even among other terrestrial isopods, *O. asellus* has relatively poor desiccation tolerance and lacks the adaptations of other species (reviewed in Hassall et al., 2018, e.g., thicker cuticles, pleopodal lungs). We therefore expected urban *O. asellus* populations to be under strong selection pressure for coping with the drier conditions in urban habitats. It is possible that, given the limitations of *O. asellus*' existing adaptations to avoid desiccation, there simply has not been enough time for increased desiccation tolerance to evolve. An alternative explanation is that while urban and rural populations do not differ in their rate of water loss and desiccation, they could differ in other aspects of water balance. Terrestrial isopods generally forage at night and need to make their water balance back in humid refugia during the day, so their survival is dependent on total water flux (Hassall et al., 2018). Our desiccation trials only measured water loss, and the humidity of their rearing environments was kept equal and high across the temperature rearing treatments, so it could be that urban and rural populations differ in their water uptake abilities through water vapor absorption (Wright & Machin, 1990, 1993), a possibility that should be investigated. The lack of divergence in desiccation tolerance between urban and rural populations could also reflect microclimate‐seeking behavior and the capacity of terrestrial isopods to locate and remain in humid environments (Edney, 1968; Hornung, 2011; Lindqvist, 1968), and to form aggregations to conserve moisture (Allee, 1926; Broly, Devigne, Deneubourg, & Devigne, 2014; Friedlander, 1965). Using behavior to buffer against variation in humidity could then lead to isopods being shielded from selection on variation in desiccation tolerance (i.e., the Bogert effect; Bogert, 1949; Muñoz & Losos, 2018; Muñoz et al., 2014). Our path analysis suggests that urban isopods could increase their desiccation tolerance by developing larger body size at higher rearing temperatures, although this was opposed by the marginally nonsignificant direct negative effect of higher rearing temperatures on desiccation tolerance. Why higher rearing temperatures may have directly reduced desiccation tolerance is an interesting question. We suspect that the 29°C rearing treatment was generally stressful, as that environmental temperature almost equaled the highest temperatures experienced in the field. The opposing effects of temperature, along with behavioral responses to variation in aridity, could explain why urban isopods have not diverged in body size.

By contrast, in other systems that show a lack of divergence in body size across urban and rural populations, other mechanisms appear to be responsible. For example, in acorn ants, the repeated lack of body size evolution across multiple urbanization gradients has been suggested to result from constraints on living within an environment of finite space, the bounds of an acorn (Yilmaz et al., 2019). More generally, evolutionary responses of body size to climatic variation across biogeographic gradients are complex across a wide array of taxa, with some studies showing the evolution of smaller body size in warmer climates, some showing no population differences, and others showing the evolution of larger body size (Atkinson & Sibly, 1997; Kingsolver & Huey, 2008). In an analogous manner, responses appear to be complex for the evolution of body size under urban heat islands (Merckx et al., 2018). Indeed, relative to other physiological, life‐history, and morphological traits, body size evolution in cities is perhaps one of the most variable in terms of consistency of the magnitude and direction of response among different cities and taxonomic groups (Diamond & Martin, 2020). Plastic effects of temperature on body size are more consistent across ectothermic species (temperature‐size rule; Kingsolver & Huey, 2008), but in our study, the pattern was opposite of the expectation under the temperature‐size rule. Isopods achieved a larger body size in warmer conditions in the laboratory, but not in the naturally warmed environment of the city. There are a number of different reasons for this result ranging from a stress response to an artificially long growing season, experiencing 29°C for nearly half a year rather than very rarely, which might have allowed isopods in the warmer rearing temperature to grow to a larger size than those reared in the cooler treatment. Given the significant plastic effects of temperature on isopod size and the effects of size on desiccation tolerance, more work is needed to uncover the mechanisms underlying these responses.

Given that *O. asellus* exhibited both predicted (heat tolerance) and surprising (desiccation tolerance) evidence for responses to urban heat islands, it is worth considering the broader literature of isopod responses to climatic variation across biogeographic gradients. Although perhaps an unsatisfying answer, it seems that the taxonomic group of isopods is especially variable in response to climatic variation in temperature and aridity. For example, they tend to be “rule‐breakers” of common biogeographic patterns such as Rapoport's rule (smaller range sizes at lower latitudes) which has been argued to arise through physiology–climate relationships (Sfenthourakis & Hornung, 2018; Stevens, 1989). Of course, individual studies of terrestrial isopods have nonetheless found support for relationships between physiological traits and biogeographic variation in climate. *Porcellio laevis*, a common terrestrial isopod, was examined for evidence of thermal adaptation to latitudinal variation in temperature. For *P. laevis*, there were no latitudinal differences in heat‐coma temperature, but there were significant increases in chill‐coma temperature and decreases in the thermal optimum for righting response speed with increasing latitude (Castañeda et al., 2004). Additionally, an interspecific study of terrestrial isopods found a significant association between desiccation tolerance (measured as water loss rate) and aridity throughout individual species distributions (Dias et al., 2013). However, notably both of these studies contrast with our results for responses of *O. asellus* to urban heat islands. We instead found supportive evidence for population divergence in heat tolerance, but not cold tolerance. And although our study focused on the indirect effect of temperature on desiccation tolerance, we still found no significant evolutionary divergence in desiccation tolerance despite substantial differences in urban versus rural habitat aridity. Whether these differences arise due to the variable nature of isopod responses to climate or to the timescale of change (i.e., long‐diverged populations and species across biogeographic clines versus recently diverged populations across urban heat islands) remains unclear.

Our study lends further support for parallel or convergent evolution of heat tolerance responses to urban heat islands. At the same time, our study highlights the variable nature of other trait responses including desiccation tolerance and body size. These traits showed no evidence of significant evolutionary divergence in response to urban heat islands while exhibiting complex plastic responses to laboratory rearing temperature. Indeed, the evolution of body size in response to urban heat islands is emerging as especially variable among different species. Admittedly, desiccation tolerance is much less studied in urban evolutionary physiology compared with thermal tolerance and body size traits, so it is difficult to assess how common the lack of evolutionary response in this trait is across different taxa. In general, our study shows how a diversity of mechanisms (plasticity and evolutionary divergence) and types of responses (from unexpected to no response to predicted) likely underlie responses to urban heat islands. Our results reinforce the fact that despite strong selection pressure, not all physiological traits will evolve rapidly in urbanized environments.

## CONFLICT OF INTEREST

None declared.

## Supporting information

Supplementary Material

## Data Availability

Data available from the Dryad Digital Repository https://doi.org/10.5061/dryad.r4xgxd297
