# Supplementary material for: Evidence for the evolution of thermal tolerance, but not desiccation tolerance, in response to hotter, drier city conditions in a cosmopolitan, terrestrial isopod
Source: Evol Appl. 2020 Aug 19;14(1):12–23. doi: 10.1111/eva.13052 (PMC7819561; doi:10.1111/eva.13052)
Supplement: Supplementary file 1 — Supplementary Material [file EVA-14-12-s001.docx]

**Supporting Information**

**Figure S1.** Diagram illustrating the two-temperature common garden rearing design

**
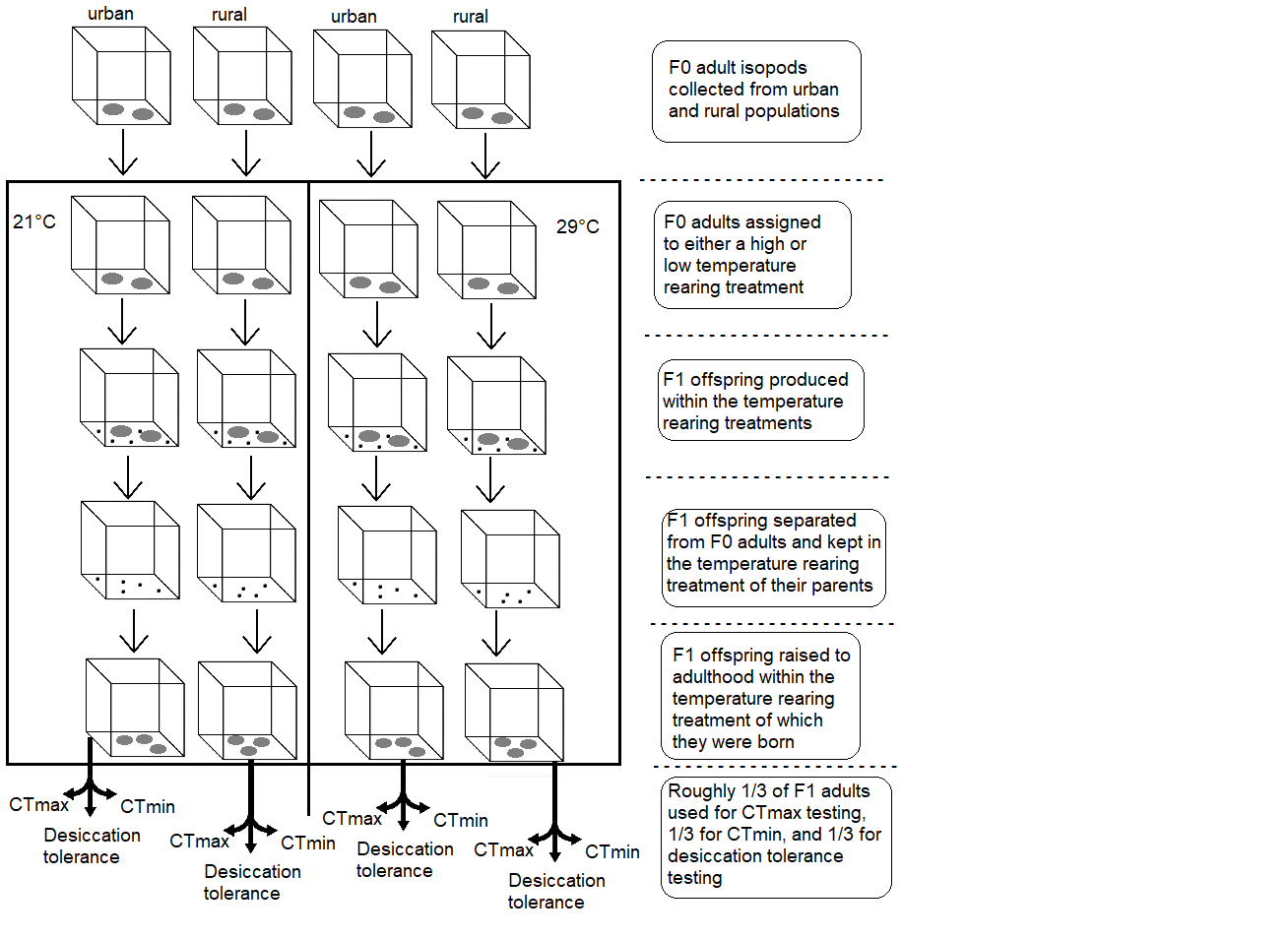
**

**Figure S2.** (a) desiccation tolerance (minutes) as a function of body mass (mg). Blue circles represent the rural population reared at the 21 °C treatment, red circles represent the rural population reared at the 29 °C rearing treatment, blue triangles represent the urban population reared at the 21 °C rearing treatment, and red triangles represent the urban population reared at the 29 °C rearing treatment. (b) Desiccation tolerance (minutes) as a function of temperature rearing treatment. Predicted values ± 1 SE from linear mixed effects models (without a covariate of body size) are shown, with the urban population in triangle symbols and the rural population in circles. (c) Desiccation tolerance (minutes) relative to body size as a function of temperature rearing treatment. Predicted values ± 1 SE from linear mixed effects models (with a covariate of body size) are shown, with the urban population in triangle symbols and the rural population in circles.

**
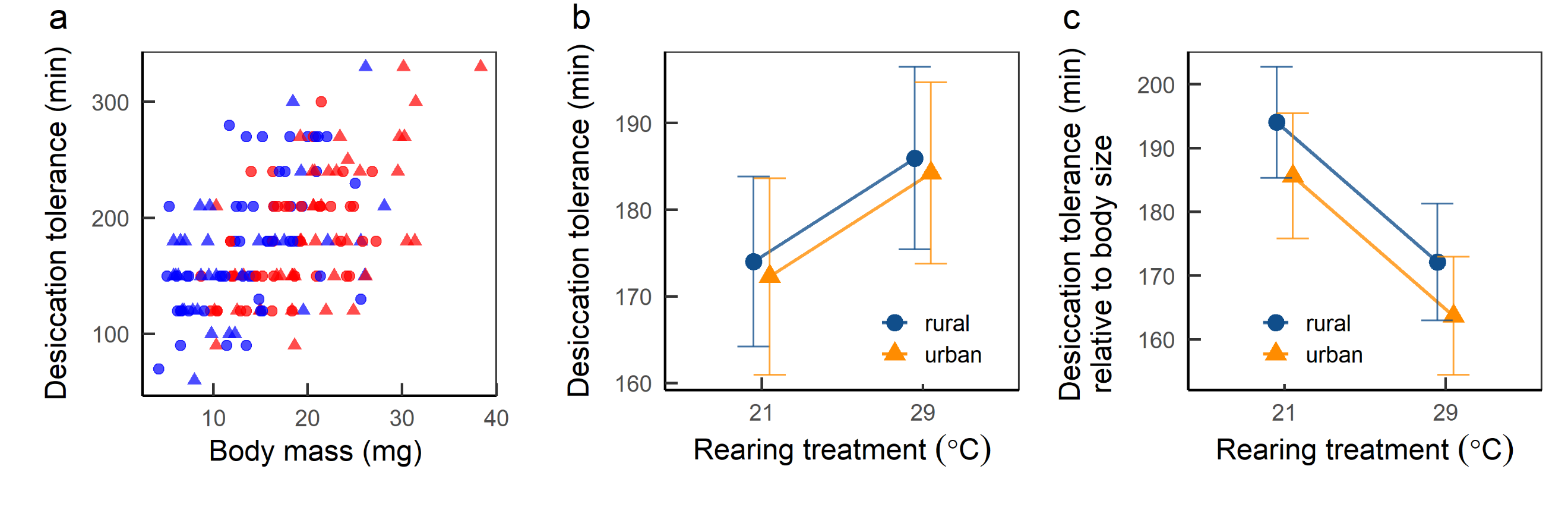
**

**Table S1.** Isopod collection sites, including their geographic coordinates (longitude and latitude), whether the replicate was sourced from an urban or rural population, the percent impervious surface area (ISA), and the number of replicates collected at each site. All sites are located in the area of Cleveland, Ohio, USA.

| Site number | Longitude | Latitude | Source population | | ISA | Number of replicates | |
| --- | --- | --- | --- | --- | --- | --- | --- |
| 1 | -81.34971 | 41.5984 | | rural | 7 | | 1 |
| 2 | -81.42791 | 41.49652 | | rural | 10 | | 1 |
| 3 | -81.42767 | 41.56013 | | rural | 16 | | 1 |
| 4 | -81.42269 | 41.42147 | | rural | 17 | | 2 |
| 5 | -81.189247 | 41.631485 | | rural | 3 | | 2 |
| 6 | -81.356416 | 41.510301 | | rural | 8 | | 2 |
| 7 | -81.342308 | 41.484845 | | rural | 5 | | 2 |
| 8 | -81.381037 | 41.440490 | | rural | 14 | | 1 |
| 9 | -81.604246 | 41.429368 | | urban | 57 | | 3 |
| 10 | -81.659057 | 41.434428 | | urban | 68 | | 2 |
| 11 | -81.71118 | 41.44457 | | urban | 94 | | 1 |
| 12 | -81.570416 | 41.511674 | | urban | 75 | | 1 |
| 13 | -81.572256 | 41.522424 | | urban | 73 | | 2 |
| 14 | -81.657930 | 41.453434 | | urban | 79 | | 1 |
| 15 | -81.564629 | 41.508393 | | urban | 75 | | 1 |
| 16 | -81.545753 | 41.506940 | | urban | 66 | | 1 |
| 17 | -81.526180 | 41.489419 | | urban | 57 | | 1 |

**Table S2.** Number of F1 individuals assayed for each trait type, rearing temperature, and source population.

| Trait | Group | | Number of individuals | |
| --- | --- | --- | --- | --- |
| Thermal tolerance | |  | | 322 |
|  | | CT_max_ assessment | | 163 |
|  | | CT_min_ assessment | | 159 |
|  | | urban source population reared at 21°C temperature | | 77 |
|  | | urban source population reared at 29°C temperature | | 75 |
|  | | rural source population reared at 21°C temperature | | 101 |
|  | | rural source population reared at 29°C temperature | | 69 |
| Desiccation tolerance | |  | | 167 |
|  | | urban source population reared at 21°C temperature | | 32 |
|  | | urban source population reared at 29°C temperature | | 43 |
|  | | rural source population reared at 21°C temperature | | 51 |
|  | | rural source population reared at 29°C temperature | | 41 |
| Body size | |  | | 491 |
|  | | urban source population reared at 21°C temperature | | 110 |
|  | | urban source population reared at 29°C temperature | | 118 |
|  | | rural source population reared at 21°C temperature | | 152 |
|  | | rural source population reared at 29°C temperature | | 111 |

**Table S3.** Number of F1 individuals per replicate assessed for desiccation tolerance, thermal tolerance and body size. Replicate designation is an arbitrary alpha-numeric descriptor to note F1 isopod groups reared together in the laboratory.

| Source population | Site number | | | Replicate designation | Number of individuals | Number assessed for desiccation tolerance | Number assessed for thermal tolerance | Number assessed for body size | |
| --- | --- | --- | --- | --- | --- | --- | --- | --- | --- |
| rural | | 1 | WAY-98 | | 26 | 9 | 17 | | 26 |
| rural | | 2 | WAY-31 | | 14 | 0 | 14 | | 14 |
| rural | | 2 | WAY-40 | | 29 | 9 | 20 | | 29 |
| rural | | 3 | WAY-201 | | 10 | 0 | 10 | | 10 |
| rural | | 4 | WAY-198 | | 9 | 9 | 0 | | 9 |
| rural | | 4 | WAY-208 | | 8 | 8 | 0 | | 8 |
| rural | | 5 | WAY-133 | | 8 | 8 | 0 | | 8 |
| rural | | 5 | WAY-220 | | 8 | 8 | 0 | | 8 |
| rural | | 6 | WAY-104 | | 29 | 9 | 20 | | 29 |
| rural | | 6 | WAY-134 | | 22 | 8 | 14 | | 22 |
| rural | | 6 | WAY-147 | | 10 | 0 | 10 | | 10 |
| rural | | 7 | WAY-182 | | 28 | 8 | 20 | | 28 |
| rural | | 7 | WAY-192 | | 20 | 0 | 20 | | 20 |
| rural | | 8 | WAY-78 | | 24 | 9 | 14 | | 24 |
| rural | | 8 | WAY-148 | | 18 | 7 | 11 | | 18 |
| urban | | 9 | WAY-68 | | 26 | 9 | 17 | | 26 |
| urban | | 9 | WAY-137 | | 16 | 0 | 16 | | 16 |
| urban | | 9 | WAY-145 | | 26 | 6 | 20 | | 26 |
| urban | | 10 | WAY-83 | | 30 | 10 | 20 | | 30 |
| urban | | 10 | WAY-92 | | 29 | 8 | 20 | | 29 |
| urban | | 11 | WAY-70 | | 10 | 0 | 10 | | 10 |
| urban | | 12 | WAY-111 | | 19 | 0 | 19 | | 19 |
| urban | | 13 | WAY-132 | | 10 | 0 | 10 | | 10 |
| urban | | 13 | WAY-142 | | 29 | 9 | 20 | | 29 |
| urban | | 14 | WAY-138 | | 8 | 8 | 0 | | 8 |
| urban | | 15 | WAY-163 | | 8 | 8 | 0 | | 8 |
| urban | | 16 | WAY-152 | | 8 | 8 | 0 | | 8 |
| urban | | 17 | WAY-153 | | 9 | 9 | 0 | | 9 |

**Table S4.** Isopod collection sites for field-caught individuals assessed for mass, including site number, their geographic coordinates (longitude and latitude), whether the replicate was sourced from an urban or rural population, the percent impervious surface area (ISA), and the number of individuals collected at each site. All sites are located in the area of Cleveland, Ohio, USA.

| Site number | Longitude | Latitude | Source population | | ISA | | Number of individuals | | |
| --- | --- | --- | --- | --- | --- | --- | --- | --- | --- |
| 2 | -81.42791 | 41.49652 | | rural | | 10 | | 40 | |
| 8 | -81.381037 | 41.440490 | | rural | | 14 | | 25 | |
| 10 | -81.659057 | 41.434428 | | urban | | 68 | | 72 |  |
| 12 | -81.570416 | 41.511674 | | urban | | 75 | | 58 |  |
| 13 | -81.572256 | 41.522424 | | urban | | 73 | | 114 |  |
| 14 | -81.657930 | 41.453434 | | urban | | 79 | | 42 |  |
| 18 | -81.300240 | 41.455704 | | rural | | 4 | | 18 |  |
| 19 | -81.294547 | 41.611271 | | rural | | 4 | | 49 |  |

**Table S5.** Comparison of two statistical model summaries for desiccation tolerance response. Estimates, standard errors, F test statistics, degrees of freedom, and *P-*values for the significance of source population, rearing temperature and the covariate of body mass are reported. Significant *P-*values at the 0.05 level are indicated in bold font.

| Model | Term | Estimate | SE | *F* | df | *P* |
| --- | --- | --- | --- | --- | --- | --- |
| Full | Body mass | 5.694 | 0.661 | 74.285 | 1,121.359 | **3E-14** |
|  | Rearing temperature | -21.894 | 11.084 | 3.902 | 1,18.441 | 0.063 |
|  | Source population | -8.442 | 10.449 | 0.653 | 1,15.336 | 0.413 |
| Reduced | Rearing temperature | 11.947 | 12.170 | 0.964 | 1,17.147 | 0.340 |
|  | Source population | -1.728 | 12.234 | 0.02 | 1,17.153 | 0.889 |

**Table S6.** Statistical model summaries for thermal tolerance and desiccation tolerance responses in response to days spent in the rearing treatment. Estimates, standard errors, F test statistics, degrees of freedom, and *P-*values for the significance of source population, rearing temperature and the covariate of body mass are reported. Significant *P-*values at the 0.05 level are indicated in bold font.
